# Acute diesel exhaust exposure and postural stability: a controlled crossover experiment

**DOI:** 10.1186/s12995-017-0182-5

**Published:** 2018-01-08

**Authors:** Jason Curran, Rachel Cliff, Nadine Sinnen, Michael Koehle, Chris Carlsten

**Affiliations:** 1 School of Population and Public Health, Faculty of Medicine, University of British Columbia, 2206 E Mall, Vancouver, BC V6T 1Z9 Canada; 2 Department of Medicine, Division of Respiratory Medicine, Chan-Yeung Centre for Occupational and Environmental Respiratory Disease, University of British Columbia, Vancouver, Canada; 3 Copeman Healthcare Centre, Suite 300 – 808 Nelson Street, Vancouver, BC V6Z 2H2 Canada; 4 School of Kinesiology, University of British Columbia, Medical Sciences Block C, Room 118, 2176 Health Sciences Mall, Vancouver, BC V6T 1Z3 Canada; 5 Vancouver General Hospital, 2775 Laurel St., 7th Floor, Vancouver, BC Canada

**Keywords:** Air pollution, Traffic, Diesel exhaust, Exposure, Balance, Postural stability, Crossover design, Bess

## Abstract

Recent epidemiological evidence connects ambient air pollutants to adverse neurobehavioural effects in adults. In animal models, subchronic controlled exposures to diesel exhaust (DE) have also showed evidence of neuroinflammation. Evidence suggests that DE not only affects outcomes commonly associated with cognitive dysfunction, but also balance impairment. We conducted a controlled human exposure experiment with 28 healthy subjects (average age = 28 years (SD = 7.1; range = 21–49); and 40% female) who were exposed to two conditions, filtered air (FA) and DE (300 μg PM2.5/m3) for 120 min, in a double-blinded crossover study with randomized exposures separated by four weeks. Postural stability was assessed by the Balance Error Scoring System (BESS), a brief, easily-administered test of static balance. The BESS consists of a sequence of three stances performed on two surfaces. With hands on hips and eyes closed, each stance is held for 20 s. “Error” points are awarded for deviations from those stances. Pre- and immediately post-exposure BESS “error” point totals were calculated and the difference between the two timepoints were compared for each of the two exposure conditions. A mixed effect model assessed the significance of the association. While our data demonstrates a trend of reduced postural stability in response to exposure to DE, exposure was not significantly associated with BESS value. This is the first study to investigate changes in postural stability as a result of exposure to DE in human subjects.

## Introduction

Traffic-related air pollution (TRAP) is a major contributor to the outdoor air pollution mix and has been heavily implicated in cardiovascular and respiratory disease [1, 2]. Emerging epidemiological evidence connects ambient air pollutants to adverse cognitive and neurobehavioural effects in adults [3]. Human studies have further demonstrated that living in areas with elevated air pollution is associated with decreased cognitive function [4–8], and elevated risk of dementia [9] and autism [10].

Controlled exposure to diesel exhaust (DE), meanwhile, has been reported to elicit a general cortical stress response in human subjects [11], elevate cytokine expression and oxidative stress in different regions of the rat brain [12], contribute to neuroinflammation and potentially lead to a rise in early markers of neurodegenerative disease [13].

Evidence suggests that DE not only affects outcomes commonly associated with cognitive deficits, such as impaired recall memory and perceptual motor speed, but is also linked to balance impairment (indicating dysfunction of vestibular, cerebellar, and associated afferent and efferent pathways for postural control) [14]. Postural stability or control represents a complex motor skill that is characterized by the ability to balance and orient the body’s position in space, and can be adversely affected by injury or disease to the vestibular system and/or brain, including Parkinson’s disease [15]. Cognitive factors are thought to play a role in the control of stability during activities such as walking and standing [16]. Additionally, environmental and occupational exposures, such as those to ambient air manganese and lead, have been reported to provoke poor postural balance [17, 18].

Human studies to date have provided limited insight into the acute effects of exposure to DE on postural stability. We conducted a crossover experiment with adult subjects who were exposed to DE under controlled settings, and hypothesized that DE inhalation would result in decreased postural stability, as assessed by the Balance Error Scoring System [19], a brief and easily-administered test of static balance. The Effects of Air Pollution on Cognition (EAPOC) study marks one of the largest human controlled DE exposure studies.

## Methods

### Subject recruitment and screening

A total of 36 study subjects were recruited via posters in or near Vancouver, B.C. transportation hubs, online notices, and e-mail notifications to the local health authority list-serve.

An initial telephone screening assessed the suitability of potential subjects according to set of inclusion and exclusion criteria that included: (i) subjects between the ages of 19 and 49; (ii) healthy; (iii) nonsmoker; (iv) speaking and reading proficiency in English; (v) not pregnant or breast-feeding; and, (vi) absence of co-existing medical conditions or medications that could interfere with the study protocol. Subjects were also excluded based on having (vii) a moderate-to-high degree of claustrophobia, and (viii) the presence of implanted metal that could interfere with functional magnetic resonance imaging (fMRI), which were considered as part of a separate component of this study not covered in this article.

A secondary screening of subjects included an in-person medical/health questionnaire and brief physical exam by our study clinician. Each qualifying subject was then presented with a detailed outline and explanation of the study protocol, and written and informed consent was obtained. Consent forms were approved by the University of British Columbia Clinical Research Ethics Board (# H12–03025), Vancouver Coastal Health Ethics Board (# V12–03025), and Health Canada’s Research Ethics Board (# 2012–0040).

### Environmental exposure procedure

Study participants were exposed to two conditions: filtered air (FA) and DE (300 μg PM_2.5_/m^3^, nominally) for 120 min, in a double-blinded, crossover study with randomized inhalation exposures separated by four weeks. Using a system previously reported [20], exposures were conducted in the Air Pollution Exposure Laboratory (APEL), which creates fresh DE, suitably aged (4 min) and diluted for human experimentation at realistic and safe concentrations. The DE dose reflects the short-term, high-ambient PM exposures commonly occurring in busy transport corridors of large cities [21], and certain occupational settings where diesel-powered machinery and generators are used [22]. The DE exposure is standardized to 300 μg PM_2.5_/m^3^, which aligns with the most common DE human health effects studies [20]. Blinding to exposure conditions was previously validated [23].

Peripheral blood was collected before and at three time points following the exposure (0-h; 3-h post-exposure; 24-h post-exposure) as part of a separate component of this study not covered in this article. Details of this blood collection procedure and serum/plasma analysis has been previously documented [24].

**Fig. 1:**
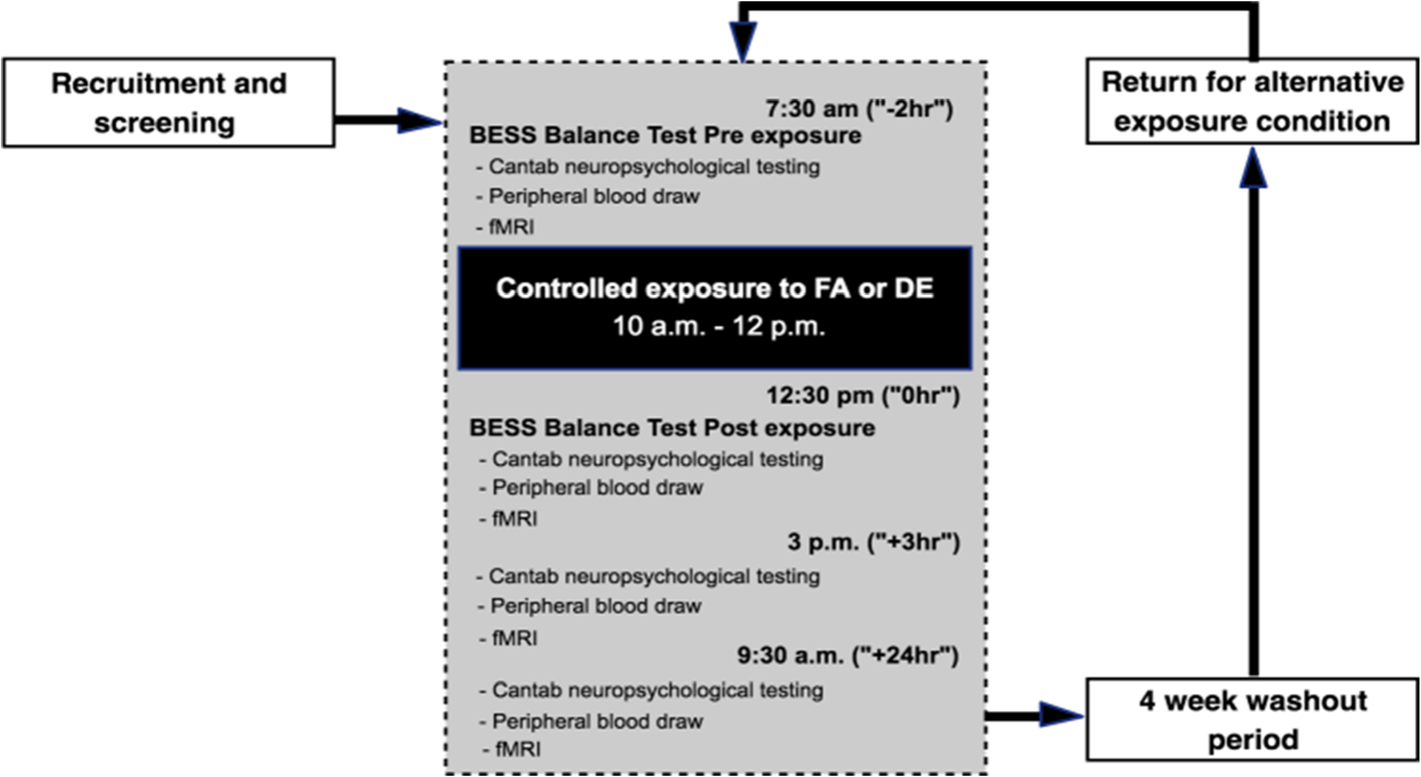
Outline of the EAPOC study design. Each crossover condition (FA/DE) was separated by a four-week washout period

During the exposure session, participants alternated between rest (40 min/h) and cycling on a stationary bike (20 min/h) at a light effort with a load set to achieve a minute ventilation of approximately 15 L/min/m^2^ body surface area. Participants were closely monitored during the 120-min exposure and vitals, including heart rate, blood pressure and peripheral oxygen saturation, were measured at 20-min intervals (Table 1 and Fig. 1).

**Table 1:** Diesel exhaust and filtered air exposure characteristics (averages and variability) from DE and FA runs in the EAPOC study

	Filtered air		Diesel exhaust	
	Mean	Standard deviation	Mean	Standard deviation
Temperature (°C)	26.6	0.6	26.4	1.2
Relative humidity (%)	32.1	8.5	35.5	7.5
PM_2.5_ concentration (μg/m^3^)	2.4	7.1	289.6	58.0
TVOC (ppb)	124.5	103.0	1425	364.5
CO_2_ (ppm)	794.1	109.0	2098	353.5
CO (ppm)	0.7	0.9	11.5	2.85
NO (ppb)	26.7	34.6	7778	2211
NO_2_ (ppb)	51.9	59.8	283.1	238.7
NO_x_ (ppb)	64.7	55.1	8062	2331

TVOC (total volatile organic compounds); PM2.5 (particulate matter aerodynamic diameter < 2.5 μm); CO_2_ (carbon dioxide); CO (carbon monoxide); NO (nitrogen oxide); NO_2_ (nitrogen dioxide); ppb (parts per billion); ppm (parts per million)

### Postural stability assessment (balance error scoring system)

**Fig. 2:**
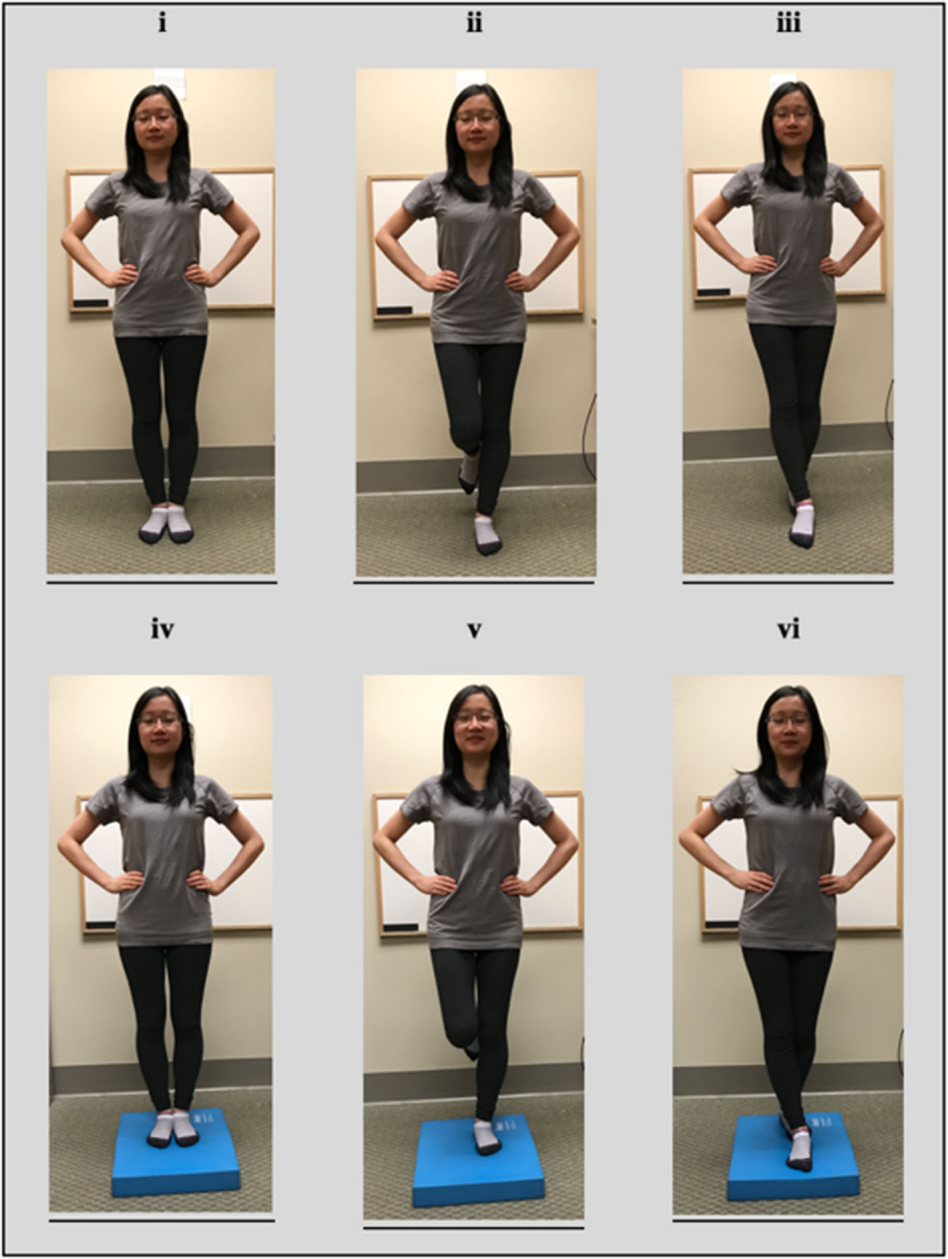
Stances used in Balance Error Scoring System (BESS): (i) double-leg stance; (ii) single-leg stance (standing on the non-dominant limb); (iii) tandem stance; (iv) double-leg stance with foam; (v), single leg on foam; and, (vi) tandem stance on foam

The BESS protocol is considered a useful test for assessing balance and one that is easy to administer [25]. A systematic review of the protocol concluded that BESS has construct validity and moderate to good reliability to assess static balance [26]. During the secondary screening and prior to data collection, each subject participated in a BESS orientation/learning session, and was also asked to maintain the same pre-test routine including the same mode of travel to the laboratory, pretest meal and caffeine intake, for each day of exposure. BESS assessments were conducted immediately prior to each randomized exposure and immediately following the exposure. Briefly, the BESS consists of a sequence of three stances (double leg stance, single leg stance, and tandem stance) performed on two surfaces (firm floor and medium density foam) [26] (Fig. 2). With hands on hips and eyes closed, each stance is held for 20 s. “Error” points are awarded for explicit deviations, including opening eyes, lifting hands off hips, abduction or flexion of the hip beyond 30 °, or stepping or stumbling. Higher score totals reflect worse performance on the test, and all tests were video recorded and scored by a blinded kinesiologist experienced in the administration of the BESS. This evaluation method has previously been shown to have an ICC of 0.88 [19].

### Statistical analysis

Statistical analysis was conducted using R (https://www.r-project.org/). Pre- and post-exposure BESS “error” point total means for each of the six stances were reported. The scores of all stances were summed for each for each exposure condition, and the difference between baseline and post-exposure aggregate scores created a delta value. Normality and order effects were tested. A mixed effect model was performed to assess the significance of the association. *P*-values of <0.05 were considered significant.

## Results

### Subject characteristics

Of the 36 recruited candidates, 28 healthy adult participants (average age = 28 years (SD = 7.1; range = 21–49); and 40% female) completed the study’s two exposure condition sessions. Complete sets of before and after exposure BESS assessments were recorded for each of the 28 participants.

### BESS assessment scores aggregated

Table 2 shows the average concentration of each BESS stance at baseline and post-exposure to DE or FA. The BESS scores for all six stances were summed, and delta values between the baseline and post-exposure timepoints were calculated for each exposure condition. Normality was assessed and the delta values were found to approximate a normal distribution. Order effects were tested by a paired *t*-test and found to be absent (*p*-value = 0.52).

**Table 2:** A summary of the BESS scores for each of the six stances and two exposure conditions. Values are presented as mean (standard deviation)

	Filtered air (FA)		Diesel exhaust (DE)	
BESS Stance	Baseline (n = 28)	Post-exposure (*n* = 28)	Baseline (n = 28)	Post-exposure (n = 28)
Double-leg stance on firm floor	0.00	0.00	0.00	0.00
Single-leg stance on firm floor	2.14 (1.98)	2.25 (1.97)	2.39 (2.45)	2.29 (2.29)
Tandem stance on firm floor	1.11 (1.69)	0.57 (0.96)	0.68 (0.94)	1.07 (1.54)
Double-leg stance on foam	0.00	0.00	0.11 (0.31)	0.04 (0.19)
Single-leg stance on foam	6.29 (2.27)	6.82 (1.94)	6.54 (1.99)	6.96 (1.93)
Tandem stance on foam	3.86 (2.01)	4.29 (2.42)	3.86 (2.41)	4.71 (2.46)
Sum of all stances	13.39 (5.81)	13.93 (5.51)	13.57 (6.37)	15.07 (5.94)

**Fig. 3:**
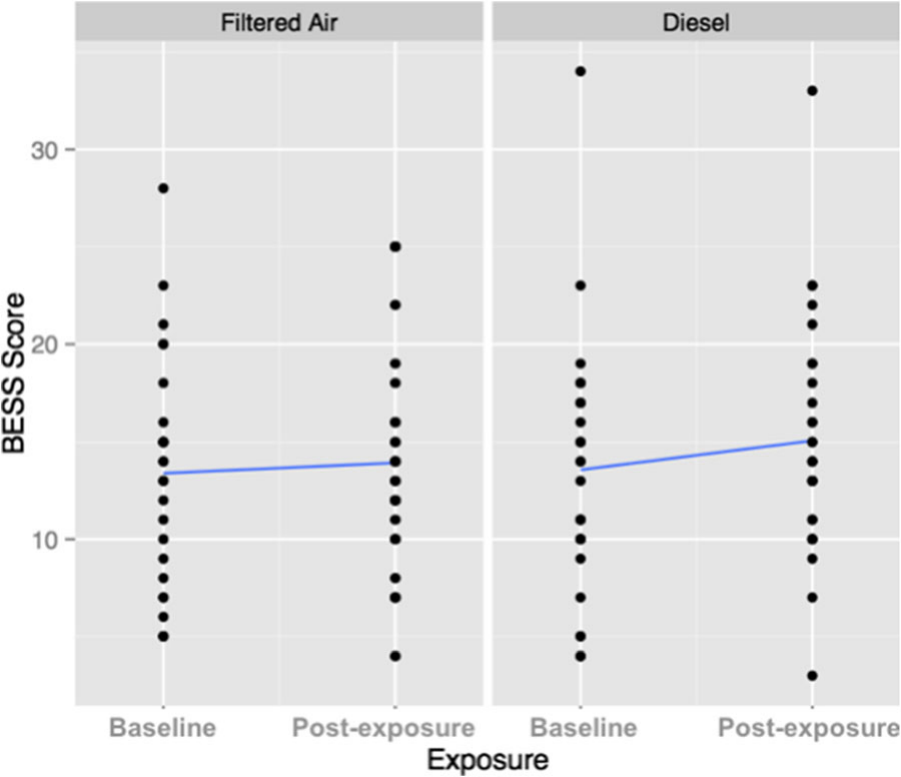
A sum of all Balance Error Scoring System (BESS) stance scores at baseline and post-exposure timepoints for sham condition (filtered air) and diesel exhaust condition

A mixed effect model assessed the significance of the association between exposure condition and the BESS delta value. In addition to the inclusion of a four-week washout period to counter potential carryover effects, our initial model evaluated the interaction between exposure and order and found no significant carryover effect (*p*-value = 0.66). Our data did demonstrate a trend of reduced postural stability in response to exposure to DE (Fig. 3). The mean change in BESS scores were greater following DE exposure (effect estimate: 1.50; 95% CI, 0.02 to 2.98), while the mean change following FA exposure was in a similar direction but was of lesser magnitude and not significant (effect estimate: 0.53; 95% CI, −0.94 to 2.01). In the mixed model, exposure did not significantly impact BESS delta score (p-value = 0.36), as suggested by the overlap in confidence intervals surrounding the estimates of effect for each exposure.

## Discussion

To our knowledge, this is the first study of its kind to investigate the effects on postural stability, or static balance, following an acute DE exposure in healthy humans. As part of the greater EAPOC study – one of the largest known human controlled exposure studies of air pollution – we hypothesized that an exposure to DE approximating 300 mg/m^3^ PM_2.5_ would negatively disrupt postural stability as measured by the BESS protocol, relative to a sham exposure. Results from our study involving 28 healthy adult subjects suggest that acute exposure to DE, with PM levels approximating those intermittently present in highly polluted cities such as Beijing, China and New Delhi, India, does not cause a significant impact to an individual’s static balance. Trends in the direction of an adverse effect (i.e. worsening balance) following DE were observed, potentially motivating more extensive or detailed examination of the role of traffic-related air pollution on factors that control postural stability. It may be that BESS scores would be further compromised at a later timepoint, assuming neuroinflammation is delayed by slow penetration of particulate matter into the central nervous system, or in a more susceptible population, such as the elderly.

In a case-control study examining the effects of chronic DE exposure in 10 railroad workers and six electricians, Kilburn found balance impairments in the DE exposed group relative to the reference group of workers [14]. However, these results are difficult to interpret as participant selection was nonrandom, potential confounding variables were incompletely controlled, and contributions likely attributable to acute versus chronic exposures were difficult to distinguish.

A limitation of our study is that our acute 2-h exposure is inconsistent with the chronic PM exposure studies to date that have found adverse associations with cognitive endpoints. While this was intentional, as we wondered about the specific effects of short-term exposures, it may be that only prolonged exposures induces sufficient inflammation to induce change across the neuro-cognitive spectrum. Furthermore, if such changes indeed exist, the BESS assessment may not be sufficiently sensitive to pick up such signals following this acute exposure. Crossover experiments typically provide greater statistical power than parallel-group trials of similar size, and allow considerably smaller sample sizes for comparable type I and type II error risks [27, 28]. Regardless, the inconclusive, but suggestive, results may motivate further investigation using a similarly designed study with a greater sample size. As suggested earlier, another limitation to our study include the recruitment of healthy adults as opposed to alternative populations who are inherently more susceptible to the CNS effects of air pollution [4, 5, 29, 30].

## Abbreviations

APEL: air pollution exposure laboratory
BESS: balance error scoring system
CNS: central nervous system
CO: carbon monoxide
CO_2_: carbon dioxide
DE: diesel exhaust
EAPOC: effects of air pollution on cognition
FA: filtered air
FMRI: functional magnetic resonance imaging
ICC: intraclass correlation coefficient
NO: nitrogen oxide
NO_2_: nitrogen dioxide
PM: particulate matter
PM_2.5_: particulate matter aerodynamic diameter less than 2.5 μm in aerodynamic diameter)
Ppb: parts per billion
Ppm: parts per million
TRAP: traffic-related air pollution
TVOC: total volatile organic compounds
